# The influence of exercise adherence on peace of mind among Chinese college students: a moderated chain mediation model

**DOI:** 10.3389/fpubh.2024.1447429

**Published:** 2024-08-30

**Authors:** Feiyang Liu, Ping Yu, Jinlong Wu, Liya Guo

**Affiliations:** ^1^School of Physical Education, Southwest University, Chongqing, China; ^2^Faculty of Psychology, Southwest University, Chongqing, China

**Keywords:** exercise adherence, meaning in life, self-control, peace of mind, self-acceptance, college students

## Abstract

**Introduction:**

Exercising adherence constitutes a pivotal approach for college students to maintain physical well-being, while peace of mind serves as a crucial indicator of their psychological health. However, few previous studies have delved into the relationship between these two variables. Our study endeavors to investigate the association between the exercise adherence and the peace of mind of college students.

**Methods:**

The Exercise Adherence Questionnaire, Meaning in Life Questionnaire, Brief Self-Control Scale, Peace of Mind Scale, and Self Acceptance Questionnaire were tested on 1,520 college students from four universities in western China, and SPSS 25.0 and Mplus 8.3 were used for analysis.

**Results:**

The results indicate a significant positive correlation between exercise adherence and meaning in life (*r* = 0.208, *p* < 0.001), self-control (*r* = 0.210, *p* < 0.001), and peace of mind (*r* = 0.237, *p* < 0.001) among college students. Mediation analysis reveals that exercise adherence has a significant direct effect on peace of mind in college students, with an effect size of 0.087. Moreover, meaning in life and self-control independently mediate the relationship between exercise adherence and peace of mind, with mediation effect sizes of 0.046 and 0.052, respectively. Additionally, meaning in life and self-control collectively exhibit a chain mediation effect, with a mediation effect size of 0.032. At the same time, the interaction terms of meaning in life and self-acceptance had a significant predictive effect on self-control (*b* = 0.090, *p* = 0.002).

**Conclusion:**

This study reveals both the relationship and intrinsic mechanisms by which exercise adherence influences the peace of mind among college students. Exercise adherence demonstrates a direct positive impact on peace of mind. Additionally, the association between exercise adherence and peace of mind is influenced by the individual mediating effects of meaning in life and self-control, as well as the chain mediation effect of meaning in life and self-control. Moreover, self-acceptance plays a positive role in regulating the relationship between meaning in life and self-control in the chain mediated pathway. This suggests that we need to encourage college students to develop the habit of exercise adherence and to carry out public welfare activities to enhance their meaning in life, self-control and self-acceptance, which will effectively promote their mental health.

## Introduction

Recently, mental health issues among Chinese university students have shown a noticeable upward trend (1). Should this trend continue, it may adversely affect the current academic and future societal development of Chinese university students. Therefore, addressing mental-health problems among Chinese university students is an urgent concern. Peace of mind refers to an individual’s inner peace and calmness, representing a form of mild positive emotion (2). Previous research has indicated that mild positive emotions are closely associated with lower levels of negative emotions and higher life satisfaction (3). Consequently, peace of mind is considered an important indicator of mental health status (4). In Eastern cultures, peace of mind is not merely a simple positive emotion but also a crucial component of subjective well-being (5). Research indicates that individuals influenced by Chinese culture tend to emphasize the pursuit of emotional well-being centered around “peace” (2). Therefore, peace of mind should be considered a pivotal focal point in enhancing the mental health of Chinese university students. In addition, the mental health problems of college students in many western countries are also increasing, and a peaceful mind may also have a positive impact on their mental health (6). However, the factors influencing university students’ peace of mind remain unclear. Hence, uncovering the influencing factors and mechanism driving of peace of mind among university students, and exploring effective ways to enhance the peace of mind of college students are not only of great significance for filling the theoretical gaps and alleviating the mental health problems of Chinese college students, but also have certain reference significance for improving the mental health problems of international college students.

One of the primary means for university students to maintain their physical and mental health is exercise adherence, which refers to long-term, regular physical exercise accomplished through investing emotion and willpower (7). Exercise adherence has been associated with numerous physical and mental benefits, including improvements in physiological function, regulation of stress responses, and reduction in depression levels (8). Although there is limited research exploring the relationship between exercise adherence and peace of mind, Fredrickson’s broaden-and-build theory of positive emotions posits that when an individual’s action patterns undergo a positive shift, the existing thought-action repertoire rises to a new level, leading to positive emotional experiences (9). Drawing on the broaden-and-build theory, exercise adherence is considered a sustained behavioral activity beneficial for both physical and mental health, representing a positive and constructive action pattern. From this perspective, university students who engage in long-term exercise are more likely to experience positive emotions. Inner tranquility is also regarded as a type of positive emotion (2); hence, exercise adherence may positively influence peace of mind. Therefore, this study hypothesizes that exercise adherence can positively influence peace of mind. Despite the potential positive impact, the specific mechanism through which exercise adherence affects peace of mind remain unclear. Consequently, this study investigates the intrinsic mechanisms through which exercise adherence influences peace of mind among university students.

For university students, meaning in life can provide them with a sense of direction and value in their lives, making it particularly crucial to their psychological and social development (10). Meaning in life refers to an individual’s perception and awareness of the essence of self and existence, among other important concepts (11). Motivational and personality-oriented theories of meaning in life propose that it arises from the ongoing process of individuals achieving self-goals (12). Based on this perspective, exercise adherence, which is a proactive and voluntary continuous behavior that demands a sustained investment of energy and attentional resources, can become a process of continuously achieving self-goals. Thus, engaging in persistent physical exercise is conducive to acquiring meaning in life (13). Moreover, physical exercise is itself considered significant for individuals’ physical and mental health development. When maintained over the long term, it can enhance an individuals’ awareness of their quality of life, subsequently aiding in strengthening their capacity to acquire meaning in life (14). As individuals deepen their understanding of life meaning during this process, their positioning of life goals becomes clearer, making them more accepting of life’s challenges in reality. Consequently, their inner satisfaction continually increases, facilitating the acquisition of peace of mind (15). Therefore, this study hypothesizes that exercise adherence may influence peace of mind through the mediating role of meaning in life.

Self-control is recognized as a crucial means to achieve self-health growth among university students (16). It refers to the conscious process in which individuals, in the absence of external supervision, intentionally overcome impulses, habits, or automatic reactions and adjust their behavior to achieve long-term goals (17). The strength model of self-control posits that the psychological energy required for self-control can be restored through positive rest (18). From this perspective, physical exercise, as a positive restorative behavior with numerous physical and mental benefits, contributes to the optimization of individuals’ subjective attitudes through long-term exercise persistence. This aids in increasing an individual’s cognitive iteration, emotional engagement, and concentration of energy (19). Consequently, this can positively affect the acquisition of psychological energy, ultimately contributing to the enhancement of self-control (20, 21). Previous research suggests that physical exercise positively affects self-control among university students (22). Furthermore, self-control is the manifestation of an individual directing attention inward, assisting in rational emotional regulation, and providing support to maintain a positive emotional state (23). Previous research has indicated that students who focus their attention inward because of increased physical exercise tend to have more positive outcomes in physical and mental health assessments. They not only exhibit lower levels of depression but also experience higher levels of inner satisfaction and peace (24). Therefore, this study hypothesized that self-control plays a mediating role in the impact of exercise adherence on peace of mind.

Although the analysis suggests that meaning in life and self-control may independently mediate the relationship between exercise adherence and peace of mind among university students, this study posits that they potentially operate in a chain-mediated manner in addition to independent factors. The “sense-making” perspective of constructivist self-development theory contends that the construction of meaning in an organism’s life is inseparable from cognition and emotion (25). According to this theory, when an individual’s meaning in life is reinforced, it aids in forming and maintaining an optimistic psychological state and a proactive psychological regulatory pattern, prompting further cognitive and emotional development (26). Cognitive and emotional development, in turn, serve as the foundation for the development of self-control (27). Therefore, enhancing meaning in life may contribute to the advancement of an individual’s self-control. Studies have found that adolescents with a strong sense of meaning in life are better able to establish and focus on long-term life goals and exhibit improved self-control when facing various temptations during the growth process (28). Building on the previously presented hypotheses, prolonged adherence to physical exercise should contribute to the formation and reinforcement of individuals’ meaning in life, which in turn assists in enhancing self-realization and self-control, ultimately promoting peace of mind. Therefore, this study further hypothesizes that meaning in life and self-control may operate in a chain-mediated fashion, through which exercise adherence influences peace of mind among university students.

Yet another aspect of peace of mind is self-acceptance, an individual’s positive attitude toward the present and future, which enables them to objectively perceive and accept their strengths and weaknesses. Self-acceptance serves as a positive facilitating factor for psychological and social development and also plays a significant role in promoting the psychological health development of university students (29, 30). The protective-protective model posits that different protective factors interact to predict individual development, where one protective factor enhances the impact of another on the outcome variable (31). According to this theory, self-acceptance and meaning in life, as protective factors in an individual’s psychological and social development, may interact with and influence self-control, which is closely associated with university students’ self-growth. Specifically, individuals with a higher degree of self-acceptance are more likely to embrace themselves and their surrounding environment unconditionally, thereby facilitating the discovery of meaning and value in their existence. This contributes to maintaining a more abundant and stable sense of meaning in life and higher levels of psychological well-being (32), subsequently assisting in the accumulation of positive psychological resources and enhancing self-control. Some studies suggest that self-acceptance is not only associated with greater meaning in life but is also linked to stronger self-control (33, 34). Additionally, self-acceptance moderates the buffering effect of meaning in life on negative emotions among university students (35). Higher levels of negative emotions are negatively correlated with individual self-control (36). This suggests that self-acceptance may expedite the positive impact of meaning in life on self-control, which is emerging as a crucial facilitating factor. Therefore, this study explored the moderating role of self-acceptance in the relationship between meaning in life and self-control.

**Figure 1:**
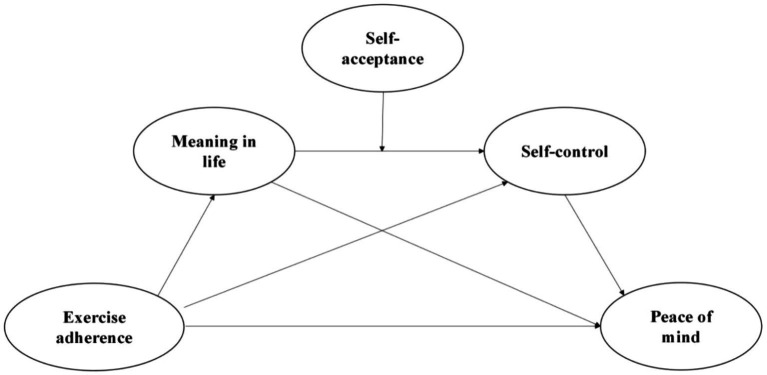
The hypothesized theoretical model.

In summary, a moderated chain mediation model was constructed to investigate, for the first time, the chained mediation effects of meaning in life and self-control on exercise adherence and peace of mind among university students. Additionally, this study explored the moderating role of self-acceptance in the mediation process (Figure 1). This study aimed to enrich the relevant theories and practices related to the mental and physical health of university students. The hypotheses of this study were as follows:

*H1*: Exercise adherence significantly positively predicts peace of mind.

*H2*: Meaning in life serves as a standalone mediator between adherence to exercise and peace of mind.

*H3*: Self-control acts as a standalone mediator between adherence to exercise and peace of mind.

*H4*: Meaning in life and self-control play chain-mediating roles in the relationship between adherence to exercise and peace of mind.

*H5*: Self-acceptance moderates the relationship between meaning in life and self-control.

## Methods

### Participants and procedures

This study used cluster random sampling to conduct a survey among first- to fourth-year undergraduate students from four universities in the western region of China. In order to enhance the power of the statistical test and reduce sampling error, a total of 1,635 questionnaires were distributed, of which 1,520 were successfully collected, resulting in a response rate of 92.966%. The participants were 634 males (41.7%) and 886 females (58.3%). The distribution of academic year variables was as follows: 432 first-year, 415 second-year, 339 third-year, and 314 fourth-year students. Regarding age distribution, the majority fell within the range of 17–25 year-olds (*M* = 20.56, *SD* = 1.58).

This study was approved by the Research Ethics Committee of Chinese Southwest University. Prior to the study, we contacted the administrators of the participating schools and obtained permission for the questionnaire test and informed consent from the students. In this study, consent was obtained from parents/legal guardians for participants under 18 years of age. All students voluntarily participated in the survey.

### Measures

#### Exercise Adherence Questionnaire

The Exercise Adherence Questionnaire (EAQ), developed by Wang et al. (7), was used to measure exercise adherence. The questionnaire consists of three dimensions–behavioral habit, effort input, and emotional experience–comprising a total of 14 items. Participants rated each item on a 5-point Likert scale, ranging from “strongly disagree” to “strongly agree,” with higher scores indicating better exercise adherence. This questionnaire has demonstrated excellent reliability in Chinese university environments (37). In this study, the Cronbach’s α coefficients for the three dimensions were 0.731, 0.744, and 0.760, respectively, while the overall Cronbach’s *α* for the entire questionnaire was 0.830. A confirmatory factor analysis (CFA) was conducted to validate the questionnaire, and the results indicated good model fit (*χ*^2^/*df* = 6.933, TLI = 0.900, CFI = 0.919, SRMR = 0.041, RMSEA =0.062).

#### Meaning in Life Questionnaire

Steger’s Meaning in Life Questionnaire (MLQ) was revised by Wang et al. (38). The questionnaire consisted of 10 items divided into two subscales: presence of meaning and search for meaning. Participants rated each item on a 7-point Likert scale, ranging from 1 (completely not applicable) to 7 (completely applicable), with higher scores indicating a stronger sense of meaning in life. The MLQ has demonstrated high reliability and validity in a Chinese school context (39). In this study, the Cronbach’s α coefficients for the MLQ’s two subscales were 0.893 and 0.925, respectively, while the overall Cronbach’s *α* for the entire questionnaire was 0.892. A validity analysis of the questionnaire yielded favorable results (*χ*^2^/*df* = 14.146, TLI = 0.950, CFI = 0.962, SRMR = 0.047, RMSEA = 0.093).

#### Brief Self-Control Scale

Self-control was measured using the Brief Self-Control Scale (BSCS) revised by Morean et al. (40). The BSCS consists of seven items and two dimensions: self-discipline and impulse control. Participants rated each item on a 5-point Likert scale, with higher scores indicating a stronger level of self-control. This scale has demonstrated high reliability and validity in a Chinese school environment (41). In this study, the Cronbach’s α coefficients for the two dimensions were 0.826 and 0.878, respectively, and the overall Cronbach’s *α* for the entire scale was 0.836. CFA results indicated good model fit (*χ*^2^/*df* = 7.396, TLI = 0.976, CFI = 0.985, SRMR = 0.026, RMSEA = 0.065).

#### Peace of Mind Scale

Peace of mind was measured using the Peace of Mind Scale (PoMS) developed by Lee et al. (2). The PoMS is unidimensional, comprising seven items rated on a 5-point Likert scale, ranging from “not at all” to “all the time.” Higher scores indicate a greater level of inner peace. This scale has demonstrated high reliability and validity in a Chinese school environment (42). In this study, the Cronbach’s *α* coefficient for the scale was 0.767, and the measurement model demonstrated good fit (*χ*^2^/*df* = 9.196, TLI = 0.920, CFI = 0.951, SRMR = 0.033, RMSEA = 0.073).

#### Self-Acceptance Questionnaire

Self-acceptance was measured using the Self-Acceptance Questionnaire (SAQ) developed by Cong and Gao (43). The SAQ comprises two dimensions: self-acceptance and self-evaluation. Responses were measured on a 5-point Likert scale, with higher scores indicating a higher level of self-acceptance. This scale demonstrated high reliability and validity in a Chinese school environment (44). In this study, the Cronbach’s *α* coefficients for the two dimensions of the scale were 0.913 and 0.868, respectively, and the overall Cronbach’s *α* was 0.907. CFA results indicated good model fit (*χ*^2^/*df* = 3.051, TLI = 0.981, CFI = 0.978, SRMR = 0.024, RMSEA = 0.037).

### Analytic strategy

The CFA was conducted using Mplus 8.3 for all questionnaires. Descriptive statistics for scores on various scales were computed using SPSS 25.0. Pearson correlation analysis in SPSS 25.0 was employed to examine the relationships between different variables. Structural equation modeling (SEM) was conducted using Mplus 8.3 to explore the mediating roles of meaning in life and self-control in the relationship between exercise adherence and peace of mind. This study also examined the moderating role of self-acceptance between meaning in life and self-control. To test the significance of the mediating effects, a bootstrap method was applied with 5,000 resampling iterations to obtain bias-corrected percentile confidence intervals for the mediation effects. A confidence interval not including zero indicates the presence of a significant mediation effect (45).

Initially, Harman’s single-factor test was used to explore the potential for common method bias. The results revealed 10 eigenvalues greater than 1 for the common factors, with the first factor accounting for 1.256% of variance. This percentage is below the critical threshold of 40% (46), suggesting the absence of a significant common method bias in this study.

### Overall discriminant validity

Mplus 8.3 was used to test the independence of five constructs: exercise adherence, meaning in life, self-acceptance, self-control, and peace of mind. The results of the independence tests are presented in Table 1. It is evident that the fit indices for the five-factor model (*χ^2^* = 85.708, *df* = 44, *χ*^2^/*df* = 1.948, TLI = 0.980, CFI = 0.987, SRMR = 0.025, RMSEA = 0.025) are significantly better than those of other models. Therefore, the overall fit of the model was considered excellent.

**Table 1:** Confirmatory factor analysis results.

Model	*χ^2^*	*df*	*χ^2^/df*	CFI	TLI	RMSEA	SRMR
Single-factor model 1	1468.891	54	27.202	0.558	0.460	0.131	0.093
Two-factor model 2	1093.734	53	20.636	0.675	0.595	0.114	0.084
Three-factor model 3	726.467	51	14.244	0.789	0.727	0.093	0.068
Four-factor model 4	575.176	48	11.983	0.835	0.835	0.085	0.062
Five-factor model 5	85.708	44	1.948	0.987	0.980	0.025	0.025

① The five variables of exercise adherence, meaning in life, self-control, peace of mind and self-acceptance were combined into one factor. ② Combining exercise adherence, meaning in life, and self-control into one factor, and merging peace of mind with self-acceptance. ③ Categorizing exercise adherence as one factor, combining meaning in life with self-control, and merging peace of mind with self-acceptance. ④ Assigning exercise adherence, meaning in life, and self-control to individual categories, while combining peace of mind with self-acceptance. ⑤ Assigning exercise adherence, meaning in life, self-control, peace of mind, and self-acceptance to their respective categories.

## Results

### Descriptive statistics among the variables

Descriptive statistics and correlational analyses were conducted for exercise adherence, meaning in life, self-control, peace of mind, and self-acceptance, as outlined in Table 2. The results indicate a predominantly significant positive correlation among the variables. Specifically, the following relationships were observed. Exercise adherence was positively correlated with meaning in life, self-control, and peace of mind (*r* = 0.212, *p* < 0.001; *r* = 0.212, *p* < 0.001; *r* = 0.243, *p* < 0.001); however, there was no correlation with self-acceptance (*r* = 0.028, *p* = 0.273). Meaning in life was positively correlated with self-control and peace of mind (*r* = 0.244, *p* < 0.001; *r* = 0.263, *p* < 0.001) but was not associated with self-acceptance (*r* = 0.027, *p* = 0.285). Self-control was positively correlated with peace of mind and self-acceptance (*r* = 0.324, *p* < 0.001; *r* = 0.169, *p* < 0.001), while peace of mind is not associated with self-acceptance (*r* = 0.044, *p* = 0.089).

**Table 2:** Descriptive statistics and correlation analysis of variables.

Variable	Mean	SD	1	2	3	4	5
1. Exercise adherence	3.841	0.594	–				
2. Meaning in life	4.796	0.935	0.212***	–			
3. Self-control	4.067	0.706	0.212***	0.244***	–		
4. Peace of mind	4.007	0.473	0.243***	0.263***	0.324***	–	
5. Self-acceptance	2.462	0.590	0.028	0.027	0.169***	0.044	–

***p < 0.001.

### Mediating role of meaning in life and self-control

A mediation analysis framework was used, focusing on the mediating variables of meaning in life and self-control. In this analysis, exercise adherence was treated as the independent variable, peace of mind as the dependent variable, and meaning in life and self-control as the mediating variables. Mediation effects were examined using a stepwise regression method, and the results are presented in Table 3. The overall fit indices for the chain mediation model are as follows: *χ^2^* = 60.429, *df* = 29, *χ*^2^/*df* = 2.084, TLI = 0.982, CFI = 0.988, SRMR = 0.026, RMSEA = 0.027. Thus, the overall model fit met the required standards.

**Table 3:** Results of mediation regression analysis.

	Peace of mind		Meaning in life		Self-control		Peace of mind	
	*b*	SE	*b*	SE	*b*	SE	*b*	SE
Exercise adherence	0.211^***^	0.027	0.444^***^	0.063	0.193^***^	0.048	0.129^**^	0.042
Meaning in life					0.264^***^	0.043	0.187^***^	0.047
Self-control							0.362^***^	0.058

**p < 0.01, ***p < 0.001.

The results indicate that exercise adherence significantly and positively influences peace of mind (*b* = 0.211, *p* < 0.001), supporting H1. When incorporating meaning in life into the regression equation, exercise adherence had a significant positive impact on meaning in life (*b* = 0.444, *p* < 0.001), and meaning in life had a significant positive impact on peace of mind (*b* = 0.129, *p* < 0.001). Thus, meaning in life partially mediated the relationship between exercise adherence and peace of mind, confirming H2. After including self-control in the regression equation, exercise adherence had a significant positive impact on self-control (*b* = 0.193, *p* < 0.001), and self-control had a significant positive impact on peace of mind (*b* = 0.362, *p* < 0.001). This suggests that self-control partially mediates the relationship between exercise adherence and peace of mind, supporting H3. Combining H2 and H3, the simultaneous regression analysis results revealed that meaning in life has a significant positive impact on self-control (*b* = 0.264, *p* < 0.001), indicating the presence of a chain mediation effect between meaning in life and self-control. Therefore, exercise adherence improves peace of mind through the chain mediation of meaning in life and self-control, supporting H4.

Table 4 presents the mediating effects of meaning in life and self-control on the relationship between exercise adherence and peace of mind. A bootstrap resampling (5,000 repetitions) was used to test the indirect effects. The results indicate that the total indirect effect between exercise adherence and peace of mind was 0.130. Specifically, there are three mediation pathways: exercise adherence → meaning in life → peace of mind (0.046), exercise adherence → self-control → peace of mind (0.052), and exercise adherence → meaning in life → self-control → peace of mind (0.032). The ratios of these three indirect effects to the total effect were 21.198, 23.963, and 14.747%, respectively. The 95% confidence intervals for all three did not include zero, indicating significance.

**Table 4:** Results of mediation analysis for meaning in life and self-control.

		Effect size	Boot CI lower limit	Boot CI upper limit	Relative effect size
Total effect		0.217	0.147	0.297	-
Direct effect		0.087	0.033	0.147	40.092%
Indirect effect	Total indirect effect	0.130	0.084	0.192	59.908%
	Indirect effect 1	0.046	0.023	0.078	21.198%
	Indirect effect 2	0.052	0.027	0.090	23.963%
	Indirect effect 3	0.032	0.017	0.057	14.747%

Indirect effect 1: exercise adherence → meaning in life → peace of mind; Indirect effect 2: exercise adherence → self-control →peace of mind; Indirect effect 3: exercise adherence → meaning in life → self-control → peace of mind.

### Testing the potential moderation effect of self-acceptance

Latent Moderation Structural Model (LMS) was used to examine the moderating effect of self-acceptance on meaning in life and self-control. To assess model fit, an initial model was constructed that included only the moderating variable without interaction terms. The results of the relevant indices indicated good model fit (*χ^2^* = 87.304, *df* = 46, *χ*^2^/*df* = 1.898, TLI = 0.987, CFI = 0.981, SRMR = 0.025, RMSEA = 0.024, Loglikelihood H0_baseline_ = −19403.073, AIC = 38894.147). Subsequently, the final model incorporating interaction terms was introduced (Loglikelihood H0_final_ = −19397.819, AIC = 38885.638).

To assess the fit of the final model, a chi-square difference test was conducted using the formula *D* = −2[Loglikelihood H0_baseline_ − Loglikelihood H0_final_]. The resulting chi-square value of 10.508 with one degree of freedom and *p* = 0.001 indicates that the final model has superior fit compared to the baseline model. The AIC values further supported the model comparison with the baseline model having a higher AIC (AIC = 38894.147) than the final model (AIC = 38885.638), reinforcing the conclusion that the final model outperformed the baseline model. Considering the baseline model’s fit, the overall analysis suggests that the fit indices of the final model meet statistical requirements. Consequently, a path coefficient analysis was conducted, as presented in Table 5.

**Table 5:** Presents the results of the moderated mediation regression analysis.

	Peace of mind		Meaning in life		Self-control		Peace of mind	
	*b*	SE	*b*	SE	*b*	SE	*b*	SE
Exercise adherence	0.211***	0.027	0.451***	0.060	0.208***	0.047	0.093**	0.027
Meaning in life					0.284***	0.040	0.105***	0.024
Self-control							0.232***	0.034
Self-acceptance					0.140***	0.020		
Meaning in life*Self-acceptance					090**	0.028		

**p < 0.01, ***p < 0.001.

**Figure 2:**
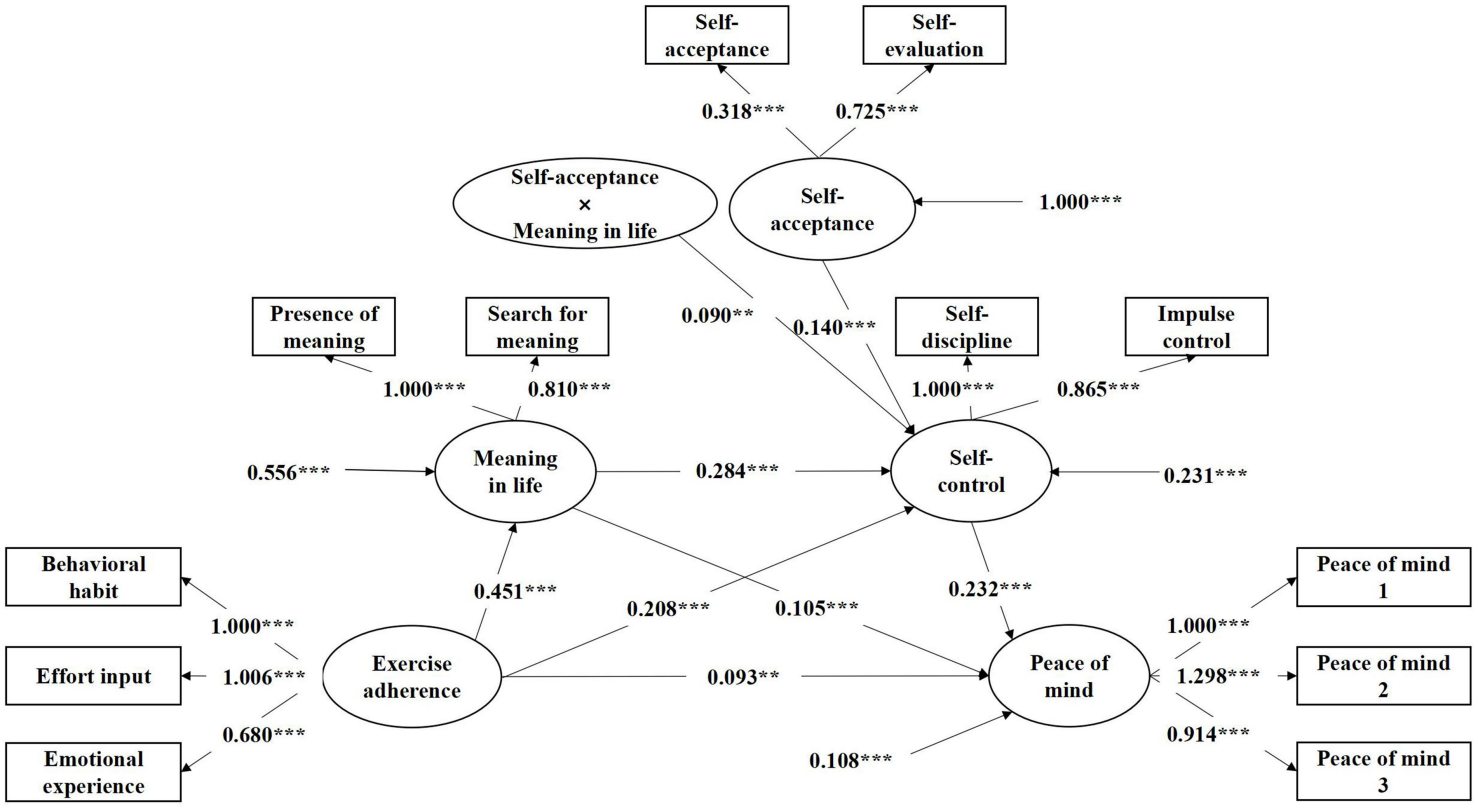
Model of the moderating mediating effect of exercise adherence on peace of mind. **p < 0.01, ***p < 0.001, significant regression coefficient.

**Figure 3:**
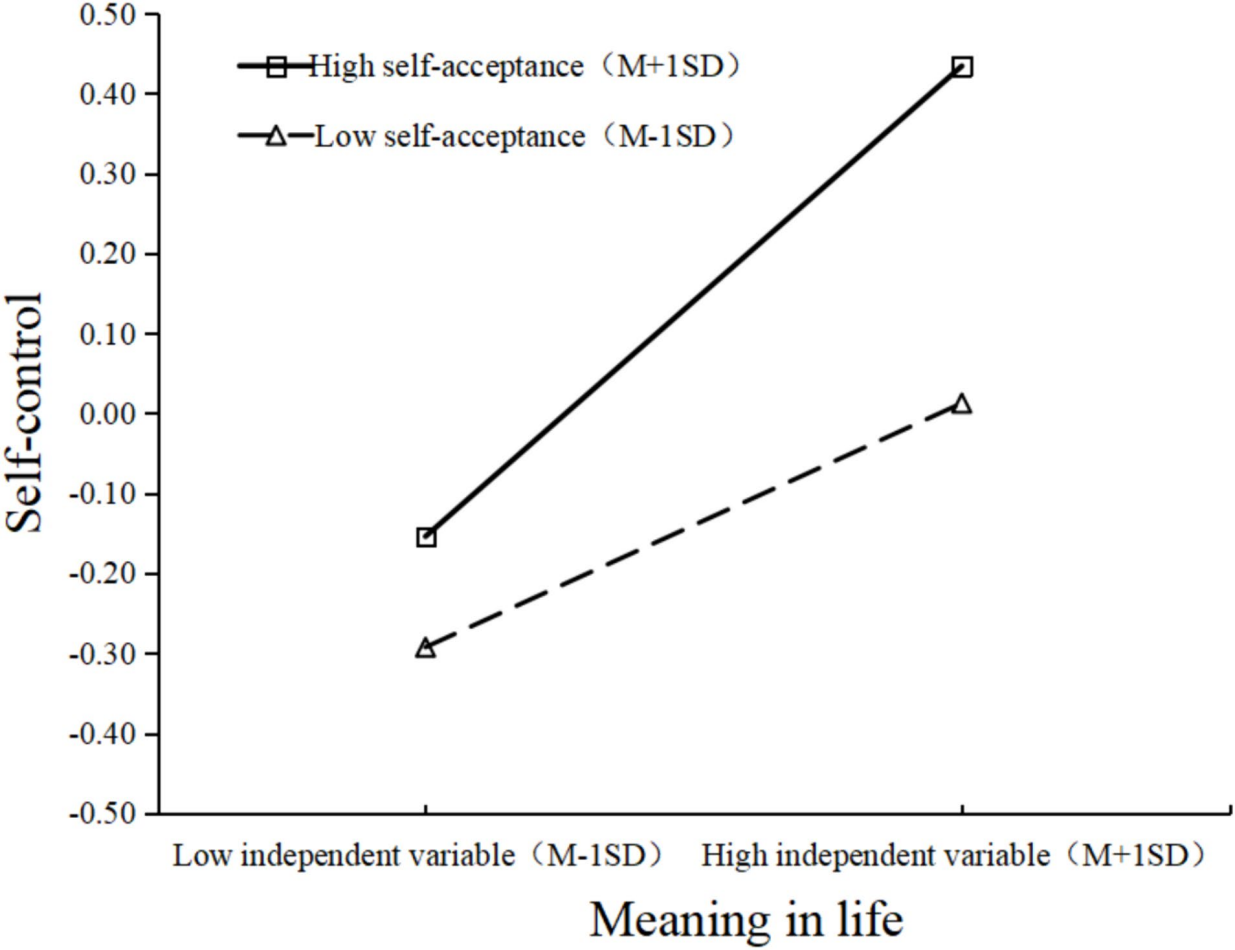
The moderating effect of self-acceptance on meaning in life and self-control.

As Table 5 shows, when self-acceptance is a moderating variable, self-control is significantly positively influenced by the interaction between meaning in life and self-acceptance (*b* = 0.090, *p* = 0.002). This indicates that self-acceptance is a significant moderating variable in the pathway from meaning in life to self-control, supporting H5. To visually depict the mechanism of the effect of exercise adherence on peace of mind and the various path coefficients, we constructed a moderated mediation model based on the model assumptions (Figure 1) and the results of the moderated mediation regression analysis (Table 5), depicted in Figure 2. Additionally, to better illustrate the moderating effect of self-acceptance, participants were divided into high- and low-acceptance groups based on one standard deviation above and below the mean, respectively. Simple slope tests were employed to examine the predictive effect of meaning in life on self-control at different moderator levels. The results are presented in Figure 3. The graph shows that when self-acceptance is low, meaning in life significantly and positively predicts self-control (*b* = 0.194, *p* < 0.001). When self-acceptance is high, meaning in life similarly significantly predicts self-control (*b* = 0.374, *p* < 0.001) but with greater impact strength.

Finally, to further examine the impact of self-acceptance on the mediating model, this study calculated changes in the size of the chain mediation effects at different levels of moderation. The analysis revealed that when self-acceptance was low, the size of the chain mediation effect (exercise adherence → meaning in life → self-control → peace of mind) is 0.020 (*p* = 0.001). In contrast, when self-acceptance was high, the chain mediation effect increase to 0.039 (*p* < 0.001). This indicates that, as the moderating variable increases, the chain mediation effect also increases.

## Discussion

This study investigated the relationship between exercise adherence and peace of mind among college students and its internal mechanisms. A moderated mediation model was constructed with meaning in life and self-control as mediating variables and self-acceptance as a moderating variable.

Initially, researchers suggested that physical exercise does not directly influence inner calmness (47). However, the results of this study indicate that exercise adherence is positively correlated with peace of mind, and consistent exercise adherence can directly predict peace of mind among college students. This implies that persistent exercise behavior is a crucial factor in deriving the physical and mental benefits of exercise. From a mind–body perspective, long-term adherence to physical exercise contributes to the continuous release of endorphins and other hormones that are closely associated with feelings of pleasure and satisfaction in the brain (48). This, in turn, leads to cumulative mental health benefits for college students (49), facilitating the attainment and improvement of peace of mind.

Furthermore, this study identified an independent mediating role for meaning in life between exercise adherence and peace of mind. The positive psychology perspective of meaning in life suggests that it lies in actively seeking a better life and life goals with a positive and optimistic attitude (12). As a core factor influencing individual subjective well-being and an important pathway to enhancing positive psychological states (50), physical exercise may bring about an optimistic mindset and positive life behaviors when consistently adhered to over the long term (51). Thus, when individuals, especially young adults, focus on the positive aspects of self-growth through consistent physical exercise, their sense of meaning in life may be enhanced. This, in turn, leads individuals to experience more meaning and fulfillment in life, reducing negative emotional factors such as perceived failures (52). Consequently, individuals’ subjective emotions tend to be more balanced and stable, contributing to peace of mind (53). Therefore, exercise adherence significantly and positively predicts meaning in life, and individuals with higher levels of meaning in life are more likely to have higher levels of peace of mind.

This study’s findings confirm that self-control independently mediates the relationship between exercise adherence and peace of mind. As an active behavior that effectively engages both the body and mind, exercise adherence requires the active involvement of both the exerciser’s body and willpower (54). This requires individuals to monitor and regulate their emotions during exercise and prepare themselves to achieve the goal of exercise adherence. Therefore, adolescents must overcome the inertia of daily life and academic pressure to maintain physical exercise. By overcoming these challenges and persisting with physical exercise, adolescents can effectively cultivate their immediate control capability and delayed gratification habits, contributing to enhancing their self-control abilities (55). As their self-control abilities improve, the infusion of positive psychological resources increases, aiding in the reversal of cognitive biases and enhancing emotional balance abilities, thus increasing peace of mind. Previous studies have also shown that individuals with good self-control are more likely to have a healthy body and mind and higher levels of subjective well-being (56, 57), which is a unique aspect of subjective well-being that is particularly important in the context of traditional Chinese culture (5). Therefore, consistent physical exercise enhances college students’ self-control and contributes to increased peace of mind, which is of particular significance for Chinese college students.

Finally, this study revealed that exercise adherence can predict college students’ peace of mind through a chain mediation effect involving both meaning in life and self-control. This finding aligns with the broaden-and-build theory of positive emotions (58), which posits that the momentary thought-action repertoire broadened by positive emotions can assist individuals in constructing enduring personal resources, leading to adaptive benefits for the present and future. In other words, increasing the positive emotions associated with exercise adherence can positively influence an individual’s future aspirations and current intentions. This not only affects cognitive levels of thinking but also enhances self-regulation capabilities in terms of emotions and behavior, thereby altering individuals’ psychological experiences. Overall, exercise adherence, a crucial source of positive emotions, can indirectly affect psychological experiences (peace of mind) by altering cognitive perceptions of the future (meaning in life) and influencing current self-regulatory mechanisms (self-control). Moreover, the value-added spiral effect proposed in the conservation of resources theory suggests that the effective utilization of one resource can promote the efficient development of other positive resources, resulting in a greater accumulation of resources (59). This concept provides a robust explanation for the chain mediation effect observed in this study. Specifically, exercise adherence can enhance college students’ meaning in life and self-control. In turn, increased meaning in life promotes higher levels of self-control, leading to a synergistic and cumulative effect that enhances resource accumulation. Together, this mechanism helps college students to elevate their peace of mind.

The moderating role of self-acceptance in the relationship between exercise adherence and peace of mind revealed in our study is noteworthy. Specifically, this moderating effect operates on the exercise adherence → meaning in life → self-control → peace of mind relationship in the chain mediation involving meaning in life and self-control. As self-acceptance strengthens, the positive connection between meaning in life and self-control also increases. In other words, the impact of exercise adherence on peace of mind through meaning in life and self-control was more significant for college students with high self-acceptance as compared to those with low self-acceptance. Within the two-dimensional subscales of meaning in life, *presence of meaning* often serves as a protective factor for individuals’ psychological well-being, while the relationship between *search for meaning* and psychological health outcomes can vary among individuals (60). Existing research suggests that engaging in meaning-seeking activities through rumination may lead to more negative outcomes (61, 62). However, individuals with strong self-acceptance are more inclined to appreciate their strengths, acknowledge and accept their weaknesses, and have a clearer understanding of the gap between their real and ideal selves. Consequently, they are more likely to progress toward their ideal selves in a constructive manner, such as through increased self-control (33), resulting in higher levels of self-control. Contrastingly, if individuals engage in excessive and passive meaning in life contemplation, it may lead to negative emotions, increased consumption of cognitive resources, and reduced levels of self-control in other domains. Therefore, as exercise adherence enhances meaning in life, college students with higher levels of self-acceptance will exhibit a more positive self-attitude. They can more clearly recognize the disparity between their ideal and real selves, reasonably assess their existing shortcomings, and prefer positive coping mechanisms, such as increasing self-control, to bring about rational changes. Consequently, they are more likely to obtain positive emotional benefits that are conducive to psychological health development and ultimately contribute to increasing peace of mind.

In summary, the findings of this study provide valuable insights into enhancing peace of mind among college students. First, at the institutional level, efforts should be made to encourage college students to participate in physical exercise by leveraging the positive effects of exercise adherence on their emotions, cognition, and attention. For example, colleges and universities can guide college students to do more physical exercise by increasing the number of hours of physical education courses and the proportion of credits of physical education courses. Moreover, educational intervention activities focusing on meaning in life, self-control, and self-acceptance should be conducted to help college students holistically enhance their peace of mind. Such as colleges and universities can carry out psychological counseling training activities focusing on exploring the meaning in life, strengthening self-control and learning self-acceptance. Second, considering that Family has a significant impact on individual growth and physical and mental health, at the family level (63), while urging college students to engage in regular physical exercise, assistance should be provided to enhance their capacity for meaning in life, self-control, and self-acceptance. Such a multifaceted approach will contribute to improving their peace of mind. For example, parents can strengthen online and face-to-face emotional communication with college students in daily life, help college students establish reasonable expectations for the future and attach importance to self-control, and view their experiences in the right way. Finally, at the individual level, college students should cultivate a consciousness of exercise adherence. They must engage in thoughtful and discerning reflection, enrich their understanding of life’s meaning, learn to focus on their strengths, accept their shortcomings, and channel their attention toward enhancing self-control. These efforts will contribute to creating favorable conditions for developing and enhancing peace of mind.

### Limitations and future directions

The present study had several limitations. First of all, due to constraints such as time and research funding, a cross-sectional research design was employed, making it challenging to establish causal relationships between the variables. Future research should consider longitudinal study designs and other methodologies to further explore these relationships. Secondly, the participants were exclusively college students, and the study did not include other student groups. Future studies could extend the research scope to explore the impact of exercise persistence on peace of mind in various student populations. Finally, there are some potential selection bias in this study. The effect of exercise adherence on peace of mind may also include other relevant variables such as emotional state and cognitive reappraisal, which need to be further explored in future studies.

## Conclusion

Our study explored the relationship between exercise adherence and peace of mind among college students and its underlying mechanisms. Exercise adherence can not only directly influences peace of mind but also exerts influence through chain mediation involving the intermediary effects of meaning in life and self-control. Furthermore, self-acceptance moderated the relationship between meaning in life and self-control through this chain mediation. Compared with college students with lower levels of self-acceptance, the influence of exercise adherence on peace of mind through meaning in life and self-control was more significant in those with higher self-acceptance levels. This not only unveils the internal psychological mechanisms through which exercise adherence affects peace of mind, but also provides theoretical support for enhancing the psychological well-being of college students from a mind–body perspective. These findings have practical implications for guiding interventions aimed at optimizing the mental health of college students.

## Data Availability

The original contributions presented in the study are included in the article/supplementary material, further inquiries can be directed to the corresponding author.
